# Performance Differences in Male Youth Basketball Players According to Selection Status and Playing Position: An Evaluation of the Basketball Learning and Performance Assessment Instrument

**DOI:** 10.3389/fpsyg.2022.859897

**Published:** 2022-05-06

**Authors:** David Rösch, Martin G. Ströbele, Daniel Leyhr, Sergio J. Ibáñez, Oliver Höner

**Affiliations:** ^1^Institute of Sports Science, Eberhard Karls University of Tübingen, Tübingen, Germany; ^2^Methods Center, Eberhard Karls University of Tübingen, Tübingen, Germany; ^3^Faculty of Sport Science, University of Extremadura, Cáceres, Spain

**Keywords:** team sports, talent identification and development, tactical skills, technical skills, diagnostic validity, reliability, systematic game observation

## Abstract

The Basketball Learning and Performance Assessment Instrument (BALPAI) has been initially developed and evaluated to assess the performance of students or youth basketball players on the entry level. As it is currently the only observational instrument that allows an overall assessment of players’ in-game performance, it might represent a valuable tool for talent identification and development purposes. To investigate this potential field of application, this study aimed to evaluate the BALPAI regarding reliability and diagnostic validity when assessing youth basketball players within a competitive setting. The study sample comprised *N* = 54 male youth players (*M*_age_ = 14.36 ± 0.33 years) of five regional selection teams (Point Guards, PG: *n* = 19; Shooting Guards and Small Forwards, SG/SF: *n* = 21; and Power Forwards and Centers, PF/C: *n* = 14) that competed at the annual U15 national selection tournament of the German Basketball Federation (*n* = 24 selected; *n* = 30 non-selected). A total of 1997 ball-bound actions from five games were evaluated with BALPAI. The inter-rater reliability was assessed for technical execution, decision making, and final efficacy. The diagnostic validity of the instrument was examined via mean group comparisons of the players’ offensive game involvement and performance regarding both selection-dependent and position-dependent differences. The inter-rater reliability was confirmed for all performance-related components (*κ*_adj_ ≥ 0.51) while diagnostic validity was established only for specific the BALPAI variables. The selection-dependent analysis demonstrated higher offensive game involvement of selected players in all categories (*p* < 0.05, 0.27 ≤ Φ ≤ 0.40) as well as better performance in shooting and receiving (*p* < 0.05, 0.23 ≤ Φ ≤ 0.24). Within the positional groups, the strongest effects were demonstrated among PG (*p* < 0.05, 0.46 ≤ Φ ≤ 0.60). The position-dependent analysis revealed that PG are more involved in total ball-bound actions (*p* < 0.05; 0.34 ≤ Φ ≤ 0.53), passing (*p* < 0.001; 0.55 ≤ Φ ≤ 0.67), and dribbling (*p* < 0.05, 0.45 ≤ Φ ≤ 0.69) compared to players in other positions. Further differences between players according to selection status and playing position were not detected. The results of this evaluation indicate that the instrument, in its current form, is not yet applicable in competitive youth basketball. The findings highlight the importance of optimizing BALPAI for reliable and valid performance assessments in this context. Future studies should investigate the application of stricter and position-specific criteria to use the observational tool for talent identification and development purposes.

## Introduction

The search for valid performance indicators in team sports such as basketball has been a focus of research and practice (Sampaio et al., 2013; Ibáñez and Feu, 2021). Multiple factors influence a player’s performance such as anthropometric, physiological, psychological, or sociological aspects as well as technical and tactical skills (Rogers et al., 2021). However, players differ regarding these factors, as players are assigned with different in-game tasks in their respective playing positions, which will be illustrated in the following by their main tasks in offense (Trninić and Dizdar, 2000; Trninić et al., 2000). In general, basketball players are categorized into five playing positions (i.e., Point Guard, Shooting Guard, Small Forward, Power Forward, and Center). *Point Guards* direct their teams’ offenses by creating and utilizing advantages through their outstanding passing and dribbling skills. *Shooting Guards* and *Small Forwards* are usually the best scorers on a team and, thus, require variable finishing skills. Additionally, Small Forwards are also capable of scoring inside and creating second-chance opportunities. *Power Forwards* and *Centers* help other players to get open by screening for them and they rebound offensively. Players in both positions operate around the basket, although Power Forwards are also capable of attacking from distance. Because of these different in-game responsibilities players’ anthropometry and physiology also vary between playing positions (Stojanović et al., 2018; Russell et al., 2021). For example, Centers are taller and heavier than Forwards or Guards enabling them to sustain contact while attacking close to the basket (e.g., Cormery et al., 2008). Although the traditional classification has been challenged as the game has evolved through rule changes and alternative classifications have been proposed (e.g., Bianchi et al., 2017; Rangel et al., 2019), it still serves as a reference point for the identification and development of youth basketball players (de la Rubia Riaza et al., 2020).

Talent identification in basketball is a complex process considering the multidimensional nature of this team sport. Coaches or scouts are usually assigned with the challenging task to identify talented players at an early stage of their athletic development and to decide on their inclusion in the respective talent development system (Johnston and Baker, 2020). To support these stakeholders in making such important decisions, it is common to assess the current performance of potential recruits by using specific testing procedures. Therefore, typically, the players’ physical and physiological skills are assessed through objective tests (Johnston et al., 2018; for a review of validated basketball-specific physical field tests see Gál-Pottyondy et al., 2021). In contrast, technical and tactical in-game performance is difficult to capture through such tests (Koopmann et al., 2020) and is thus mainly evaluated based on the “coach’s eye”—an intuitive, subjective, experience-based, and holistic evaluation (Lath et al., 2021). For example, the National Basketball Association (NBA) employs these approaches at the annual draft combine to support the teams evaluating prospective players (Teramoto et al., 2018; Cui et al., 2019; García-Rubio et al., 2020). The combine consists of a series of measurements of anthropometric and physiological parameters as well as an assessment of the players’ shooting skills. In addition, scrimmages are organized where the coaches subjectively evaluate the players regarding their technical and tactical skills. To date, little has been published about this common way of players’ in-game evaluation and the possibilities to support such practice with objective data (Roberts et al., 2019; Höner et al., 2021). Complementary data collection could help to standardize this process by confirming coaches’ subjective impressions or, conversely, to improve the accuracy of talent identification systems by contradicting them (Baghurst et al., 2021; Johnston et al., 2021).

For match analysis in basketball, four methods of game observation are distinguished—*subjective impression analysis*, *scouting*, *qualitative game observation*, and *systematic game observation* (König and Heckel, 2021). Among these, systematic game observation is the only method that acquires quantitative data applying specific criteria, while the others rely mainly on qualitative data. At every basketball game on international level, systematic game observation is used to gather game-related statistics (e.g., points, assists, and turnovers) which are therefore conveniently utilized to inform selection decisions (Butterworth et al., 2013). Extensive research on such data revealed performance indicators that discriminate between basketball teams (e.g., Lorenzo et al., 2010; García et al., 2014). For example, differences between winning and losing teams can be identified based on field goals and defensive rebounds (García et al., 2013). Further, differences between senior players depending on their playing position were demonstrated in such performance data, although they have been studied less frequently (Sampaio et al., 2006; Choi et al., 2015; Escudero-Tena et al., 2021).

Game-related statistics are also recorded in elite youth basketball and recent studies analyzed the data for position-dependent differences. García-Rubio et al. (2019) examined performance differences of Point Guards, Shooting Guards, and Centers competing in the Adidas Next Generation Tournament (ANGT, U18). The results show that Point Guards recorded more assists than both Shooting Guards and Centers. However, Shooting Guards registered more rebounds than Point Guards and more 3-point shot attempts than Centers. Centers had more 2-point shot attempts, rebounds, and blocks than Point Guards, whereas they were not better than Shooting Guards in any of the categories examined. Kokanauskas et al. (2021) identified multiple position-dependent differences in the game-related statistics of the U16, U18, and U20 European youth championships held in 2016/17, 2017/18, and 2018/19. For example, Point Guards were found to have more minutes, assists, turnovers, and steals compared to other playing positions. Further, such performance data have been shown to predict future success of youth basketball players in adulthood (Berri et al., 2011; Rösch et al., 2021), whereas selection-dependent differences in game-related statistics remain to be analyzed in basketball-related research. However, within such data, only the efficacy of a play action (i.e., action specific to the game of basketball) is considered, while other performance-related components like decision making or technical execution are excluded. For example, a youth player’s shooting performance is thereby only evaluated by made or missed baskets, disregarding that he might also make good shooting decisions and execute them well technically. Thus, these components (i.e., decision making and technical execution) provide valuable information for selection decisions or the development of individual training recommendations. Moreover, they could contribute to an even more reliable prediction of future success (Schorer et al., 2020).

Instead of solely recording the output of play actions (i.e., game-related statistics) during systematic game observations, observational tools may be used that focus on the process of executing play actions while considering these components. The Game Performance Assessment Instrument (GPAI; Oslin et al., 1998) and the Team Sport Assessment Procedure (TSAP; Gréhaigne et al., 1997) are the most commonly used instruments for the assessment of tactical game performance in all team sports (Arias and Castejón, 2012; Barquero-Ruiz et al., 2020). Based on these instruments, specific tools have been developed for various team sports. For basketball, the Basketball Offensive Game Performance Instrument (BOGPI; Chen et al., 2013) was developed to evaluate the offensive game performance competency of preservice teachers in basketball. It assesses a player’s offensive game performance while dribbling, passing, and shooting with respect to technical skill execution, decision making, and support. However, the BOGPI is not validated for youth basketball and only assesses few offensive actions using a binary rating scale without evaluating the efficacy of a play action. The Individual Technical-Tactical Basketball Performance Assessment Instrument (IAD-BB; Folle et al., 2014) analyzes offensive and defensive actions of players in formative developmental stages regarding adaptation, decision making, and efficacy. Hatem et al. (2020) employed this instrument in Brazilian youth basketball and were able to identify position-dependent differences between Guards, Forwards, and Centers in the ball-bound actions of shooting, dribbling, and receiving. However, the IAD-BB does not consider the technical execution of a play action and is currently only available in Portuguese. The Basketball Learning and Performance Assessment Instrument (BALPAI; Ibanez et al., 2019) is the latest observational tool and has been initially developed and evaluated to assess the performance of students or youth basketball players on the entry level in small-sided basketball games (i.e., 3 vs. 3). It allows the assessment of players’ participation in offensive and defensive play actions, as well as an analysis of their respective performance in these actions according to decision making, technical execution, and final efficacy. The instrument has been utilized to demonstrate the progress of students in basketball lessons applying different teaching methodologies in primary education in Spain (González-Espinosa et al., 2017, 2019) and an adapted version of the BALPAI was successfully employed in soccer lessons (García-Ceberino et al., 2020). The BALPAI advances with respect to the differentiated evaluation of play actions (e.g., shooting or passing) on a three-point scale according to specific criteria. As it is currently the only observational instrument that allows an overall assessment of players’ in-game performance, it might represent a valuable tool for talent identification and development purposes in competitive youth basketball. However, it is unknown so far whether the instrument can be applied in such a context as it was not designed and validated for this purpose.

The aim of the present study was to evaluate the BALPAI when applied to youth basketball players within a competitive national selection tournament. This was pursued by two objectives: *First*, the inter-rater reliability was assessed for all performance-related components of the instrument. *Second*, the diagnostic validity of the observational tool was examined via selection-dependent and position-dependent differences in BALPAI variables. In talent identification research, it is common to examine differences in performance variables between more and less skilled athletes, assuming that usually higher performing athletes are selected for talent development purposes (Johnston et al., 2018). Thus, it was expected that selected players perform better than non-selected players regarding the investigated BALPAI variables. More specifically, in a first step, diagnostic validity was evaluated with respect to selection-dependent differences by testing the following hypotheses:

*H1a*: Selected players outperform non-selected players with respect to offensive game involvement and performance in shooting, passing, dribbling, receiving, and total ball-bound actions.

*H1b*: *Within* each positional group, selected players outperform non-selected players regarding offensive game involvement and performance in shooting, passing, dribbling, receiving, and total ball-bound actions.

Moreover, the playing positions differ regarding their specific requirements (Trninić and Dizdar, 2000; Trninić et al., 2000). Based on these requirements, it was expected that players in certain playing positions would perform better than those in other positions regarding specific BALPAI variables. Thus, in a second step, the diagnostic validity was examined by analyzing position-dependent differences. For this purpose, the following hypotheses were derived from the positional requirements:

*H2a*: Point Guards are more involved in passing, dribbling, and total ball-bound actions than both Shooting Guards and Small Forwards as well as Power Forwards and Centers.

*H2b*: Point Guards perform better in passing and dribbling actions than both Shooting Guards and Small Forwards as well as Power Forwards and Centers.

*H2c*: Shooting Guards and Small Forwards demonstrate higher offensive game involvement and performance in shooting actions compared to Point Guards as well as Power Forwards and Centers.

Additionally, based on the evaluation of the BALPAI in terms of reliability and diagnostic validity, implications for the optimization of the observational instrument were derived.

## Materials and Methods

### Sample

The present study was conducted at the annual U15 national selection tournament of the German Basketball Federation (Deutscher Basketball Bund, DBB). This event represents the first stage of talent selection at the national level in Germany and involved eight regional selection teams with a total of 96 players. Each team played three games in this tournament. In all games, the official rules of the International Basketball Federation (FIBA) were applied with exception of a shorter total playing time (i.e., two 15-min halftimes). The coaches of the youth national teams observed these games and selected 40 players for further talent development purposes.

Five regional selection teams were chosen for this study according to the final standings of the tournament with two higher-ranked teams, two lower-ranked teams, and one team in between being considered. Thus, a balanced choice was maintained regarding the performance level of the teams. The investigated players were members of these regional selection teams and they also competed in the highest German U16 league (Jugend Basketball Bundesliga, JBBL).

Out of 96 male youth basketball players involved in the tournament, *N* = 54 players (*M*_age_ = 14.36 ± 0.33 years) comprise the sample of the present study. From these players, *n* = 24 (*M*_age_ = 14.26 ± 0.39) were selected by the federation while *n* = 30 (*M*_age_ = 14.43 ± 0.26) were not. The proportion of selected players in the sample (44.44%) was comparable to that in the tournament (41.67%). No significant difference in chronological age was detected when comparing selected and non-selected players, *t*(52) = 1.92, *p* = 0.06. Consequently, influences related to chronological age (e.g., relative age effect; de la Rubia Riaza et al., 2020) were not further considered in this study. The number of players at each playing position both in total and separated by their selection status is displayed in Table 1.

**Table 1:** Total number of players at each playing position separated by selection status.

Playing position	Total (*N* = 54)	Selected (*n* = 24)	Non-selected (*n* = 30)
	*n*		
Point Guard	19	9	10
Shooting Guard	10	5	5
Small Forward	11	4	7
Power Forward	11	4	7
Center	3	2	1

For analyses purposes, players were assigned to three positional groups (PG: *n* = 19; SG/SF: *n* = 21; and PF/C: *n* = 14) based on the similarity of their in-game responsibilities (Trninić and Dizdar, 2000; Trninić et al., 2000).

### Procedures

One out of three games from each of the sampled teams was randomly selected for analysis in this study. Overall, a total of 1997 ball-bound actions from five games were evaluated with the BALPAI. All games were retrospectively analyzed by one coder. To evaluate the coding procedure (Objective 1), a subset of one game with a total of 498 ball-bound actions (24.94% of all actions) was additionally rated by a second independent coder. The first coder who rated all games was 28 years old, a licensed basketball coach and an experienced basketball player for 8 years. The second coder was 25 years old, completed a basic and major subject in basketball during his studies and played recreational basketball. In line with notational analyses in other team sports (e.g., Muñoz et al., 2018), both coders were trained in the use of the instrument: Initially, they were provided with general information about the study’s objectives and design. Subsequently, the coders were trained with video samples and exemplary ratings after they had been familiarized with the rating system and the assessment criteria. Afterward, both coders had to rate 15 min of a competitive basketball game that did not involve any of the players examined in the present study. Finally, the researchers and coders met to discuss questions and specific game situations where the coders disagreed with each other or rated comparable scenes differently themselves.

The games were filmed by the German Basketball Federation (Deutscher Basketball Bund, DBB) and publicly shared through an online platform (see Supplementary Table 2). The videos and team rosters were additionally provided to the researchers by the DBB. The selection status of the players (selected or non-selected), as well as the playing positions determined by the respective clubs (Point Guard, PG; Shooting Guard, SG; Small Forward, SF; Power Forward, PF; and Center, C), was obtained through an online search (Deutscher Basketball Bund, 2020; NBBL gGmbH, 2020). All data processed in this study were publicly available. The analyses were based solely on secondary data, and the aggregated values did not allow conclusions to be drawn about individuals. The university’s ethics department and the DBB approved the implementation of this study.

### Measures

The games were analyzed using the Basketball Learning and Performance Assessment Instrument (Ibanez et al., 2019). It is designed to assess both offensive and defensive play actions, but it also allows to focus only on certain items (Ibanez et al., 2019, p. 7). Thus, only ball-bound actions in offense performed in the frontcourt were considered in this study (i.e., shooting, dribbling, passing, and receiving). For these actions, both the performance of the players and their participation were assessed. Performance was evaluated with respect to three components (i.e., decision making, technical execution, and final efficacy). Within these components, each play action was rated according to its adequacy (i.e., adequate = 3 points, neutral = 2 points, and inadequate = 1 point). The ratings were conducted according to the instrument’s assessment criteria, which are exemplified by the evaluation of a player’s decision making in shooting (see Annex 1; Ibanez et al., 2019). Making a shot was rated (a) adequate when there was no clear defensive pressure and when the condition to shoot was more favorable than that of the teammates, (b) neutral when there was clear defensive pressure or there was a teammate in a more favorable condition to shoot, and (c) inadequate when there was clear defensive pressure and there was a teammate in a more favorable condition to shoot. Based on the specific ratings for each play action, three performance indices were computed with respect to the performance-related components (PI_DM_, PI_TE_, and PI_FE_) and additionally compiled in a Total Performance Index (PI):


$$\begin{array}{l} Performance\;Index\;for\;Decision-Making\;\left({\text{PI}}_{\text{DM}}\right)=\frac{\begin{array}{c} \text{Sumof points for}\\ \text{decision-making} \end{array}}{\begin{array}{c} \text{Total ball-bound actions}\\ \text{performedbyaplayer in offense} \end{array}} \end{array}$$

$$\begin{array}{l} Performance\;Indexfor\;Technical\;Execution\;\left({\text{PI}}_{\text{TE}}\right)=\frac{\begin{array}{c} \text{Sumof points for}\\ \text{technical execution} \end{array}}{\begin{array}{c} \text{Total ball-bound actions}\\ \text{performedbyaplayer in offense} \end{array}} \end{array}$$

$$\begin{array}{l} Performance\;Index\;for\;Final\;Efficacy\;\left({\mathrm{PI}}_{\mathrm{FE}}\right)=\frac{\begin{array}{c} \mathrm{Sum}\mathrm{of points}\\ \mathrm{for final efficacy} \end{array}}{\begin{array}{c} \mathrm{Total ball-bound actions performed}\\ \mathrm{by}a\mathrm{player in offense} \end{array}} \end{array}$$

$$Total\;Performance\;Index\;\left(\text{PI}\right)=\frac{{\text{PI}}_{\text{DM}}+{\text{PI}}_{\text{TE}}+{\text{PI}}_{\text{FE}}}{3}$$

Moreover, a score for match participation is usually calculated that reflects the involvement of a player in both offensive and defensive actions (Ibanez et al., 2019). Considering that only offensive actions were evaluated in this study, Offensive Game Involvement (OGI) was used as an alternative score. Due to differences in playing style, the sampled teams varied regarding the total number of ball-bound actions (see Supplementary Table 1). Therefore, to compare the involvement of the players in the game regardless of their teams’ playing style, the data for each player were normalized with respect to the total ball-bound actions of each team:


OffensiveGameInvolvement(OGI, %)=Total ball-bound actions performedbyaplayer in offenseTotal ball-bound actions performedbyhisteam in offense×100

The performance scores (i.e., PI, PI_DM_, PI_TE_, and PI_FE_), as well as the game involvement (i.e., OGI), were computed and analyzed for all ball-bound actions accumulated, but also with respect to each category (i.e., shooting, dribbling, passing, and receiving).

### Statistical Analysis

The data were analyzed using IBM SPSS Version 27.0 (IBM Corporation, Armonk, NY, United States). Additionally, a web-based PABAK-OS calculator was utilized for the reliability analyses (Vannest et al., 2016). The alpha level for significance was set at *p* < 0.05.

With respect to the first objective, the inter-rater reliability was assessed using Cohen’s weighted kappa *κ*_w_ (Cohen, 1968). However, imbalances were detected in the marginal distributions of the observed ratings that could lead to possible prevalence problems (Hallgren, 2012). Therefore, the prevalence-adjusted bias-adjusted kappa *κ*_adj_ (Byrt et al., 1993) adapted for ordinal scaled data (PABAK-OS) was additionally computed. Besides, the total percentages of agreement were reported. All kappa values were interpreted according to Landis and Koch (1977).

Regarding the second objective, the investigation of the distributional properties revealed that the distributions of the data deviated from the assumption of a normal distribution, with the exception of only four variables. Considering these assumptions and the small sample sizes of the respective groups, non-parametric analyses were conducted to examine the diagnostic validity of the BALPAI. As it was hypothesized that selected players are better than non-selected players with respect to the BALPAI variables, one-tailed Mann–Whitney *U*-tests were performed to examine selection-dependent differences overall (H1a) and within the positional groups (H2b). Kruskal–Wallis tests with *post-hoc* pairwise comparisons (Bonferroni adjusted Mann–Whitney *U*-tests) were conducted to identify position-dependent differences. To evaluate the diagnostic validity of the BALPAI, one-tailed *post-hoc* tests were conducted for those group comparisons where hypotheses about position-dependent differences in offensive game involvement and performance could be derived in advance (H2a–H2c). However, for several comparisons, no hypotheses could be established based on the respective positional requirements. Although not used for diagnostic validation, two-tailed *post-hoc* tests were performed for these comparisons to provide a comprehensive analysis of position-dependent differences. Additionally, effect sizes ω and Φ were calculated and classified according to Cohen (1992).

## Results

### Reliability (Objective 1)

With respect to the first objective, Cohen’s weighted kappa indicated moderate agreement between the raters for decision making (*κ*_w_ = 0.48), fair agreement for technical execution (*κ*_w_ = 0.39), and almost perfect agreement for final efficacy (*κ*_w_ = 0.81). However, according to PABAK-OS, the inter-rater reliability for decision making (*κ*_adj_ = 0.51) was found to be moderate, while those for technical execution (*κ*_adj_ = 0.86) and final efficacy (*κ*_adj_ = 0.87) were almost perfect. The total percentages of agreement were 67.47% for decision making, 90.36% for technical execution, and 91.37% for final efficacy.

### Diagnostic Validity (Objective 2)

#### Selection-Dependent Differences

Table 2 displays the descriptive statistics and effect sizes for the BALPAI variables separated by selection status and playing position.

**Table 2:** Descriptive statistics and effect sizes for BALPAI variables separated by selection status and playing position.

BALPAI variables	Total			PG			SG/SF			PF/C		
	Selected (*n* = 24)	Non-selected (*n* = 30)		Selected (*n* = 9)	Non-selected (*n* = 10)		Selected^a^ (*n* = 9)	Non-selected (*n* = 12)		Selected (*n* = 6)	Non-selected (*n* = 8)	
	*M* ± *SD*		Φ	*M* ± *SD*		Φ	*M* ± *SD*		Φ	*M* ± *SD*		Φ
**All actions**												
OGI (%)	10.64 ± 5.04	6.90 ± 3.63	0.37^**^	13.77 ± 4.83	8.96 ± 4.00	0.51^*^	9.70 ± 4.44	6.45 ± 3.15	0.33	7.37 ± 3.96	5.00 ± 2.81	0.24
PI (pts)	2.70 ± 0.09	2.67 ± 0.11	0.12	2.71 ± 0.08	2.73 ± 0.11	0.21	2.74 ± 0.07	2.66 ± 0.08	0.51^*^	2.62 ± 0.07	2.61 ± 0.12	0.09
PI_DM_	2.55 ± 0.16	2.46 ± 0.22	0.19	2.58 ± 0.11	2.57 ± 0.20	0.15	2.61 ± 0.15	2.46 ± 0.20	0.42^*^	2.41 ± 0.15	2.33 ± 0.21	0.28
PI_TE_	2.90 ± 0.09	2.89 ± 0.10	0.06	2.92 ± 0.05	2.92 ± 0.07	0.09	2.92 ± 0.05	2.87 ± 0.10	0.22	2.83 ± 0.15	2.86 ± 0.11	0.07
PI_FE_	2.66 ± 0.12	2.66 ± 0.12	0.09	2.64 ± 0.13	2.68 ± 0.12	0.07	2.71 ± 0.12	2.66 ± 0.09	0.11	2.62 ± 0.07	2.64 ± 0.17	0.17
**Shooting**												
OGI (%)	11.69 ± 7.44	6.09 ± 4.55	0.40^**^	13.94 ± 8.38	5.02 ± 5.21	0.60^**^	10.87 ± 7.95	7.24 ± 4.82	0.25	9.54 ± 5.02	5.69 ± 3.24	0.35
PI (pts)	2.47 ± 0.23	2.36 ± 0.29	0.24^*^	2.44 ± 0.25	2.22 ± 0.28	0.48^*^	2.55 ± 0.14	2.44 ± 0.22	0.28	2.40 ± 0.31	2.40 ± 0.35	0.07
PI_DM_	2.23 ± 0.33	2.05 ± 0.48	0.19	2.16 ± 0.41	1.83 ± 0.38	0.47^*^	2.36 ± 0.28	2.22 ± 0.53	0.08	2.16 ± 0.26	2.08 ± 0.47	0.00
PI_TE_	2.86 ± 0.21	2.88 ± 0.23	0.11	2.85 ± 0.23	2.95 ± 0.11	0.27	2.94 ± 0.08	2.81 ± 0.29	0.23	2.77 ± 0.28	2.88 ± 0.23	0.33
PI_FE_	2.31 ± 0.31	2.14 ± 0.56	0.19	2.31 ± 0.20	1.87 ± 0.69	0.46^*^	2.35 ± 0.36	2.28 ± 0.27	0.04	2.25 ± 0.41	2.25 ± 0.64	0.03
**Passing**												
OGI (%)	10.17 ± 5.56	7.33 ± 4.47	0.27^*^	14.06 ± 4.18	11.06 ± 4.86	0.35	9.09 ± 5.19	6.07 ± 2.61	0.36^*^	5.95 ± 4.54	4.58 ± 3.23	0.17
PI (pts)	2.79 ± 0.14	2.80 ± 0.17	0.13	2.82 ± 0.08	2.83 ± 0.13	0.25	2.84 ± 0.11	2.74 ± 0.19	0.31	2.66 ± 0.17	2.85 ± 0.18	0.54
PI_DM_	2.85 ± 0.21	2.79 ± 0.21	0.20	2.89 ± 0.09	2.83 ± 0.15	0.19	2.90 ± 0.12	2.80 ± 0.15	0.37^*^	2.72 ± 0.38	2.73 ± 0.34	0.02
PI_TE_	2.87 ± 0.14	2.83 ± 0.23	0.03	2.88 ± 0.12	2.89 ± 0.14	0.06	2.88 ± 0.12	2.72 ± 0.27	0.30	2.83 ± 0.21	2.94 ± 0.18	0.46
PI_FE_	2.65 ± 0.26	2.78 ± 0.25	0.30	2.70 ± 0.18	2.79 ± 0.12	0.28	2.75 ± 0.27	2.70 ± 0.33	0.02	2.43 ± 0.24	2.88 ± 0.22	0.71
**Dribbling**												
OGI (%)	11.01 ± 6.65	6.65 ± 4.40	0.36^**^	16.22 ± 5.96	10.11 ± 4.70	0.50^*^	9.17 ± 5.66	6.09 ± 2.84	0.33	5.97 ± 3.18	3.15 ± 2.76	0.45^*^
PI (pts)	2.76 ± 0.12	2.70 ± 0.24	0.10	2.74 ± 0.11	2.74 ± 0.21	0.09	2.80 ± 0.08	2.71 ± 0.18	0.46^*^	2.74 ± 0.18	2.65 ± 0.36	0.07
PI_DM_	2.77 ± 0.16	2.74 ± 0.36	0.13	2.75 ± 0.12	2.76 ± 0.37	0.33	2.80 ± 0.10	2.71 ± 0.35	0.02	2.76 ± 0.29	2.75 ± 0.39	0.06
PI_TE_	2.91 ± 0.12	2.90 ± 0.20	0.11	2.94 ± 0.06	2.97 ± 0.11	0.18	2.96 ± 0.05	2.90 ± 0.15	0.18	2.79 ± 0.18	2.82 ± 0.31	0.29
PI_FE_	2.61 ± 0.22	2.48 ± 0.41	0.20	2.53 ± 0.20	2.51 ± 0.26	0.15	2.65 ± 0.15	2.52 ± 0.26	0.37^*^	2.66 ± 0.34	2.37 ± 0.69	0.24
**Receiving**												
OGI (%)	10.30 ± 4.48	7.12 ± 3.82	0.34^**^	11.17 ± 4.18	7.78 ± 3.89	0.36	10.53 ± 4.56	6.64 ± 4.18	0.41^*^	8.66 ± 5.12	6.70 ± 3.53	0.09
PI (pts)	2.70 ± 0.13	2.63 ± 0.17	0.23^*^	2.72 ± 0.09	2.64 ± 0.16	0.32	2.73 ± 0.12	2.65 ± 0.19	0.24	2.63 ± 0.17	2.59 ± 0.17	0.09
PI_DM_	2.24 ± 0.24	2.10 ± 0.29	0.24^*^	2.24 ± 0.19	2.15 ± 0.16	0.20	2.32 ± 0.24	2.09 ± 0.39	0.41^*^	2.12 ± 0.31	2.07 ± 0.26	0.05
PI_TE_	2.94 ± 0.11	2.91 ± 0.16	0.05	2.98 ± 0.07	2.89 ± 0.21	0.30	2.94 ± 0.08	2.97 ± 0.08	0.27	2.88 ± 0.17	2.86 ± 0.16	0.09
PI_FE_	2.92 ± 0.10	2.87 ± 0.19	0.06	2.95 ± 0.07	2.87 ± 0.22	0.11	2.93 ± 0.11	2.90 ± 0.20	0.01	2.88 ± 0.13	2.84 ± 0.17	0.11

*OGI, Offensive Game Involvement; PI, Total Performance Index; PI_DM_, Performance Index for Decision Making; PI_TE_, Performance Index for Technical Execution; PI_FE_, Performance Index for Final Efficacy; PG, Point Guard; SG/SF, Shooting Guard and Small Forward; and PF/C, Power Forward and Center*.

a *One player did not take any shot. Thus, PI, PI_DM_, PI_TE_, and PI_FE_ were not calculated and the sample size was reduced to *n* = 8 for these performance indicators*.

* *p* *< 0.05;*

** *p < 0.01*.

With respect to selection-dependent differences (H1a), Mann–Whitney *U*-tests demonstrated higher offensive game involvement in all categories for selected players compared to non-selected players (192.50 ≤ *U* ≤ 244.50, *p* < 0.05). Hereby, medium effect sizes were found in all categories (0.34 ≤ Φ ≤ 0.40) except for involvement in passing, where a small effect size was detected (Φ = 0.27). Moreover, selected players showed a better shooting performance (*U* = 246.00, *p* < 0.05) and outperformed non-selected players in receiving and specifically in decision making regarding this action (260.00 ≤ *U* ≤ 262.50, *p* < 0.05). Small effect sizes were revealed for all these performance-related differences (0.23 ≤ Φ ≤ 0.24). No performance advantages were detected for selected players in passing (*U* ≤ 346.00, *p* ≥ 0.08), dribbling (*U* ≤ 318.00, *p* ≥ 0.07), or total ball-bound actions (*U* ≤ 336.50, *p* ≥ 0.09).

The results of the Mann–Whitney *U*-tests within the three positional groups (H1b) revealed that selected PG were significantly more involved in shooting (*U* = 11.00, *p* < 0.01), dribbling (*U* = 18.50, *p* < 0.05), and total ball-bound actions (*U* = 18.00, *p* < 0.05). Thereby, strong effect sizes were found in all categories (0.50 ≤ Φ ≤ 0.60). Further, selected PG demonstrated a better shooting performance as well as a better decision making and final efficacy regarding this action (19.50 ≤ *U* ≥ 20.50, *p* < 0.05) than non-selected players on this position. Medium effect sizes were found for all these performance-related differences (0.46 ≤ Φ ≤ 0.48). Selected SG/SF were significantly more involved in passing (*U* = 30.50, *p* < 0.05) and receiving (*U* = 27.50, *p* < 0.05). Medium effect sizes were demonstrated for the differences in both categories (0.36 ≤ Φ ≤ 0.41). Moreover, performance-related differences were found for dribbling (*U* = 24.50, *p* < 0.05) and overall game performance (*U* = 21.00, *p* < 0.05). Specifically, selected players showed a better decision making in passing (*U* = 30.50, *p* < 0.05), receiving (*U* = 27.50, *p* < 0.05), and total ball-bound actions (*U* = 27.00, *p* < 0.05) than non-selected players on these positions. Further, they demonstrated a better final dribbling efficacy (*U* = 30.50, *p* < 0.05). A strong effect size was revealed for the difference in overall game performance (Φ = 0.51), while medium effect sizes were found for the other performance-related differences among players on these positions (0.37 ≤ Φ ≤ 0.46). Selected PF/C displayed a higher involvement in dribbling actions with a medium effect size (*U* = 11.00, *p* < 0.05, Φ = 0.45). No further expected differences between players within the respective positional groups were identified.

#### Position-Dependent Differences

Table 3 displays the descriptive statistics and multiple group comparisons for the BALPAI variables separated by playing position.

**Table 3:** Descriptive statistics and multiple group comparisons for BALPAI variables separated by playing position.

BALPAI variables	Descriptive statistics				Kruskal–Wallis Test		*Post-hoc* analyses^a^		
	Total (*N* = 54)	PG (*n* = 19)	SG/SF^b^ (*n* = 21)	PF/C (*n* = 14)			PG vs. SG/SF	PG vs. PF/C	SG/SF vs. PF/C
	*M* ± SD				H(2)	*ω*	Φ		
**All actions**									
OGI (%)	8.56 ± 4.66	11.23 ± 4.95	7.84 ± 4.01	6.02 ± 3.43	10.36^**^	0.44	**0.34** ^*^	**0.53** ^*^	0.23
PI (pts)	2.68 ± 0.10	2.72 ± 0.10	2.70 ± 0.09	2.61 ± 0.10	9.32^**^	0.42	0.19	0.48^*^	0.40
PI_DM_	2.50 ± 0.20	2.58 ± 0.16	2.52 ± 0.19	2.36 ± 0.18	9.02^*^	0.41	0.19	0.49^*^	0.37
PI_TE_	2.89 ± 0.09	2.92 ± 0.06	2.89 ± 0.09	2.84 ± 0.12	3.75	0.26	0.18	0.31	0.19
PI_FE_	2.66 ± 0.19	2.66 ± 0.13	2.68 ± 0.10	2.63 ± 0.14	0.23	0.06	0.01	0.04	0.10
**Shooting**									
OGI (%)	8.58 ± 6.58	9.25 ± 8.11	8.80 ± 6.44	7.34 ± 4.38	0.18	0.06	**0.03**	0.03	**0.08**
PI (pts)	2.40 ± 0.27	2.32 ± 0.28	2.48 ± 0.20	2.40 ± 0.32	3.30	0.25	**0.29**	0.18	**0.08**
PI_DM_	2.13 ± 0.43	1.99 ± 0.42	2.28 ± 0.44	2.11 ± 0.38	5.62	0.32	**0.35** ^*^	0.20	**0.24**
PI_TE_	2.87 ± 0.22	2.90 ± 0.18	2.86 ± 0.24	2.84 ± 0.25	0.84	0.12	**0.12**	0.14	**0.03**
PI_FE_	2.21 ± 0.47	2.08 ± 0.56	2.31 ± 0.30	2.25 ± 0.53	1.76	0.18	**0.20**	0.17	**0.01**
**Passing**									
OGI (%)	8.59 ± 5.14	12.48 ± 4.68	7.36 ± 4.11	5.16 ± 3.75	19.36^***^	0.60	**0.55** ^***^	**0.67** ^***^	0.25
PI (pts)	2.79 ± 0.15	2.83 ± 0.10	2.78 ± 0.17	2.77 ± 0.19	0.45	0.09	**0.09**	**0.10**	0.01
PI_DM_	2.82 ± 0.21	2.85 ± 0.13	2.84 ± 0.14	2.73 ± 0.34	0.40	0.09	**0.05**	**0.10**	0.08
PI_TE_	2.85 ± 0.19	2.88 ± 0.13	2.79 ± 0.23	2.89 ± 0.19	4.55	0.29	**0.20**	**0.27**	0.31
PI_FE_	2.72 ± 0.26	2.74 ± 0.16	2.72 ± 0.30	2.69 ± 0.32	0.12	0.05	**0.06**	**0.00**	0.03
**Dribbling**									
OGI (%)	8.59 ± 5.88	13.00 ± 6.05	7.41 ± 4.44	4.36 ± 3.17	18.83^***^	0.59	**0.45** ^*^	**0.69** ^***^	0.37
PI (pts)	2.73 ± 0.20	2.74 ± 0.17	2.75 ± 0.15	2.69 ± 0.29	0.11	0.04	**0.03**	**0.04**	0.05
PI_DM_	2.75 ± 0.29	2.75 ± 0.27	2.75 ± 0.27	2.75 ± 0.34	0.50	0.10	**0.06**	**0.09**	0.10
PI_TE_	2.91 ± 0.16	2.95 ± 0.09	2.93 ± 0.12	2.81 ± 0.25	2.51	0.22	**0.05**	**0.25**	0.23
PI_FE_	2.53 ± 0.34	2.52 ± 0.23	2.58 ± 0.23	2.49 ± 0.57	0.64	0.11	**0.14**	**0.07**	0.00
**Receiving**									
OGI (%)	8.53 ± 4.39	9.38 ± 4.29	8.31 ± 4.67	7.71 ± 4.19	1.40	0.16	0.13	0.20	0.05
PI (pts)	2.66 ± 0.15	2.68 ± 0.14	2.69 ± 0.16	2.60 ± 0.16	2.96	0.23	0.01	0.26	0.26
PI_DM_	2.16 ± 0.28	2.19 ± 0.17	2.19 ± 0.35	2.09 ± 0.27	2.36	0.21	0.04	0.26	0.20
PI_TE_	2.92 ± 0.14	2.93 ± 0.16	2.96 ± 0.08	2.86 ± 0.16	4.84	0.30	0.01	0.32	0.33
PI_FE_	2.90 ± 0.16	2.90 ± 0.17	2.92 ± 0.16	2.86 ± 0.15	2.64	0.22	0.09	0.20	0.26

*OGI, Offensive Game Involvement; PI, Total Performance Index; PI_DM_, Performance Index for Decision Making; PI_TE_, Performance Index for Technical Execution; PI_FE_, Performance Index for Final Efficacy; PG, Point Guard; SG/SF, Shooting Guard and Small Forward; and PF/C, Power Forward and Center*.

a *Group comparisons referring to the diagnostic validation of the BALPAI (Objective 2; H2a–H2c) were performed utilizing one-tailed *post-hoc* tests. In these cases, effect sizes were printed in bold. For the remaining comparisons, two-tailed *post-hoc* tests were performed to provide a comprehensive analysis of position-dependent differences*.

b *One player did not take any shot. Thus, PI, PI_DM_, PI_TE_, and PI_FE_ were not calculated and the sample size was reduced to *n* = 20 for these performance indicators*.

* *p* *< 0.05;*

** *p* *< 0.01;*

*** *p < 0.001*.

With respect to position-dependent differences, Kruskal–Wallis tests revealed significant differences between positional groups regarding the involvement in passing [H(2) = 19.36, *p* < 0.001], dribbling [H(2) = 18.83, *p* < 0.001], and total ball-bound actions [H(2) = 10.36, *p* < 0.01]. Hereby, strong effect sizes were demonstrated for the involvement in passing and dribbling (0.59 ≤ *ω* ≤ 0.60), while a medium effect size was found for the involvement in total ball-bound actions (*ω* = 0.44). The *post-hoc* analyses revealed that PG are significantly more involved in passing (all *p* < 0.001), dribbling (PG vs. SG/SF: *p* < 0.05; PG vs. PF/C: *p* < 0.001), and total ball-bound actions (all *p* < 0.05) compared to players in other positions (H2a). Thereby, the comparison of PG and SG/SF revealed a strong effect size for involvement in passing (Φ = 0.55) as well as a medium effect size for involvement in dribbling (Φ = 0.45) and total ball-bound actions (Φ = 0.34). Strong effect sizes were found for these variables comparing PG and PF/C (0.53 ≤ Φ ≤ 0.69).

However, Kruskal–Wallis tests identified no significant differences between positional groups for involvement in shooting [H(2) = 0.18, *p* = 0.91]. Moreover, no performance-related differences were found in passing [H(2) ≤ 4.55, *p* ≥ 0.80], dribbling [H(2) ≤ 2.51, *p* ≥ 0.29], or shooting [H(2) ≤ 5.62, *p* ≥ 0.06]. Consequently, the *post-hoc* analyses did not confirm the expected performance advantages for PG in passing (PG vs. SG/SF: *p* ≥ 0.31; PG vs. PF/C: *p* ≥ 0.18) and dribbling (PG vs. SG/SF: *p* ≥ 0.58; PG vs. PF/C: *p* ≥ 0.23) when compared to SG/SF and PF/C (H2b). Likewise, no higher offensive game involvement in shooting was found for SG/SF (H2c; SG/SF vs. PG: *p* = 0.29; SG/SF vs. PF/C: *p* = 0.96). Moreover, no performance advantages regarding this action were found for SG/SF in comparison with PG (*p* ≥ 0.10) or PF/C (*p* ≥ 0.25), except for SG/SF outperforming PG in decision making with a medium effect size (Φ = 0.35, *p* < 0.05).

## Discussion

The present study aimed to evaluate the applicability of the BALPAI in competitive youth basketball. It examines a highly selective sample within an ecologically valid setting and is among the first to analyze process-oriented performance data of competitive youth basketball players. The use of such data for talent identification purposes has only been sporadically studied across team sports (Schorer et al., 2020). Therefore, this study contributes to a research gap by evaluating a promising observational tool regarding the investigated objectives. The inter-rater reliability for all performance-related components was confirmed (Objective 1), whereas diagnostic validity was established only for specific BALPAI variables (Objective 2). The selection-dependent analysis revealed that selected players were more involved in ball-bound actions and performed better than non-selected players in shooting and receiving. Within the positional groups, the strongest effects were found among PG. The position-dependent analysis showed higher offensive game involvement of PG in total ball-bound actions, passing and dribbling compared to players in other positions. Further differences between players according to selection status and playing position were not detected.

### Reliability (Objective 1)

The first objective of this study was to evaluate the inter-rater reliability to ensure that differences in BALPAI variables reflect actual differences in players’ performance and not random measurement errors (Schweizer et al., 2020). During the reliability analyses, a prevalence problem was detected with respect to technical execution (see Table 3). Due to the high performance level of the players, many actions were rated with the highest possible score (i.e., three points). This resulted in imbalanced marginal distributions of the observed ratings and unrepresentatively low values of Cohen’s weighted kappa (Hallgren, 2012). Therefore, PABAK-OS and the total percentages of agreement were additionally reported. Considering all coefficients, the analyses revealed satisfactory results, indicating almost perfect agreement between the raters for technical execution and final efficacy as well as moderate agreement with respect to decision making. In the original study designing and validating the BALPAI, almost perfect agreement between raters was found regarding all three components assessing the performance of fifth-grade students (Ibanez et al., 2019). However, in the present study, elite youth basketball players competing in the national selection tournament of the German Basketball Federation were assessed. In this context, it should be acknowledged that the estimates of inter-rater reliability might be substantially reduced when a rating system is applied to a new population due to restrictions of range of talent (Hallgren, 2012; Ackerman, 2014). Further, it should be noted that the applied criteria were designed for small-sided basketball games (i.e., 3 against 3). However, tactical decisions within regular basketball games (i.e., 5 against 5) are more complex due to the increased number of players. For example, in the format 3 against 3, a player in possession of the ball has only two options to pass the ball to an open teammate (i.e., two other players on his team). However, in a regular 5 against 5, the players’ options are doubled. Thus, discrepancies may have occurred in the evaluation of players’ decision making when applying the criteria in the present study.

### Diagnostic Validity (Objective 2)

#### Selection-Dependent Differences

Regarding the second objective, the diagnostic validity of the BALPAI was initially evaluated by analyzing selection-dependent differences in the assessed data. It was hypothesized that selected players would outperform non-selected players with respect to offensive game involvement and performance.

The results confirm higher *offensive game involvement* for selected players in all categories supporting the diagnostic validity of the BALPAI (H1a). Previously, selection-dependent differences in youth basketball players have mainly been investigated with respect to physical performance parameters (e.g., anthropometry, Torres-Unda et al., 2013). Thus, the comparison of the results with those of other studies is difficult. However, the findings of the current study with respect to offensive game involvement correspond with those found in other youth team sports. For example, Saward et al. (2019) reported that male youth soccer players retained by an academy in England performed more dribbles in matches between Premier League Academies compared to those released. Further, Schorer et al. (2020) found that the reached league level in adulthood of female youth handball players in Germany is determined by the number of actions taken but not the quality of those actions. The findings of these studies suggest that youth players in these team sports who are more involved in the respective game have higher chances for short-term (e.g., selection) and long-term success (e.g., performance level in adulthood). This is also indicated by the results of the present study with respect to short-term success.

*Performance-related differences* in the current study were only detected in shooting and receiving. Therefore, diagnostic validity was not established for most of the BALPAI variables in this context. Guimarães et al. (2019) found better shooting, passing, and dribbling skills in male youth players selected for an elite regional team in Portugal compared to their non-selected counterparts. With respect to shooting, the results of the present study confirm the findings of Guimarães et al. (2019). However, technical skills were assessed through basketball-specific tests in this study. Given the simplified conditions in such tests (e.g., no defending players), it may have been possible to discriminate between players in more categories than in the present study, in which players’ performance was assessed in a real basketball game. Further, the analysis of the game-related statistics from international tournaments of youth and senior national teams demonstrated that players on winning teams performed better in shooting than those on losing teams (Csataljay et al., 2009; Lorenzo et al., 2010; Milanovic et al., 2016; Leicht et al., 2017). Studies on national team programs demonstrated that at least 70% of youth basketball players selected for such programs were retained from one year to the next (Kalén et al., 2021). Moreover, players that were members of a senior national team in Europe played three international youth championships on average in their careers (Kalén et al., 2017). In this context, the importance of shooting skills is further emphasized for players who aim to get selected for such programs and want to contribute to youth and senior national teams’ success. However, no comparable studies were found on performance in receiving. Previous research reported that the performance in skills prior to shooting (e.g., receiving the ball) may affect shooting effectiveness (Okazaki et al., 2015). Therefore, selected players who perform better in receiving may also be better in shooting.

However, the diagnostic validity was not established regarding performance-related differences in passing, dribbling, or total ball-bound actions. The reason for that might be that the evaluation criteria have been developed for students or youth basketball players on the entry level (Ibanez et al., 2019). In the given competitive context, these criteria were applied to elite youth basketball players. Therefore, also the performance of non-selected players has been rated quite high. For example, this is particularly evident in the ratings for technical execution of total ball-bound actions performed by non-selected players (PI_TE_ = 2.89 ± 0.10; see Table 2). A ceiling effect was detected in this performance-related variable, as the non-selected players averaged almost the highest possible rating (i.e., three points). Moreover, the youth national team coaches may have followed a different selection pattern. Thus, players may have been selected who did not perform well in the examined games or even in the tournament, but who the coaches expect to perform best in the long term (Trunić and Mladenović, 2014; Buekers et al., 2015). This could have affected the mean performance indicators compared in this study.

To the best of our knowledge, there are no comparable studies investigating performance differences between selected and non-selected players within different playing positions in youth basketball. However, one goal of talent identification decisions is to identify developing athletes with the potential to become successful performers in adulthood (Till and Baker, 2020). In team sports such as basketball, the individual performance of the players is linked to the respective team’s success. Thus, studies are referred that analyzed within-position differences in the performance of high performing senior players of winning and losing teams. Hence, the game-related statistics of successful senior basketball players were compared to see if they are already reflected in the performance data of selected youth basketball players in the same playing positions. Further, the results are discussed according to the positional requirements.

Selected PG were more involved in shooting, dribbling, and total ball-bound actions (H1b). Further, they outperformed non-selected players on this position with respect to shooting. The central role of the point guard in a basketball teams’ attack has been confirmed for youth and senior basketball by in-depth analyses of passing sequences (Clemente et al., 2015; Korte and Lames, 2018). The results of the present study reflect this centrality as selected players are more involved in their teams’ offensive game play. Further, previous research in senior basketball found that PG from winning teams score more points with higher efficiency from all distances than those from losing teams (Choi et al., 2015; Escudero-Tena et al., 2021). However, PG are usually less responsible for scoring points but more for directing the offense by dribbling the ball and passing it to their teammates (Trninić and Dizdar, 2000; Trninić et al., 2000). In this context, the results indicate that also in elite youth basketball, the PG has to take responsibility for scoring besides organizing the game (Bianchi et al., 2017).

Selected SG/SF were more involved in passing and receiving while they outperformed non-selected players on these positions with respect to dribbling and overall game performance. It is also noticeable that selected SG/SF made better decisions in total ball-bound actions, passing, and receiving. These findings are also consistent with those found in the analyses of position-dependent differences in players’ game-related statistics on winning and losing teams. Escudero-Tena et al. (2021) found more assists in both SG and SF while Choi et al. (2015) emphasized that both Guards and Forwards contributed positively to victory by providing more assists and fewer turnovers. While more assists can be associated with the higher number of passes and better decision making executing these actions, fewer turnovers can be linked to both better passing decisions and better dribbling performances. However, both studies also reiterated the importance of scoring for players in these playing positions. In contrast, the findings of the present study suggest that selected SG/SF are not primarily expected to score points to get selected. The descriptive statistics even show a tendency for PG being slightly more involved in shooting actions while SG/SF being only the second option in this regard (see Table 3). Instead, they have to separate themselves from non-selected players by their versatility, making smart decisions with the ball and involving their teammates. Rangel et al. (2019) highlighted the high degree of versatility among players in these positions, which is generally shown by players accomplishing multiple tactical demands.

Selected PF/C only displayed a higher involvement in dribbling actions. However, players in these positions are generally assigned to help other players to get open (e.g., by screening for them) instead of creating by themselves (Trninić and Dizdar, 2000; Trninić et al., 2000). Therefore, the results suggest that the youth national team coaches were looking for players in these positions who are capable to create (e.g., their own shot) off the dribble. This conclusion is also supported by the findings of the position-dependent analysis in this study, which revealed that PF/C have fewer ball-bound actions than players on other positions (see Table 3). Thus, when they got the ball, they should use this chance to create off the dribble in order to get selected. However, research has reported that players on winning teams in these positions deliver more assists (Choi et al., 2015; Escudero-Tena et al., 2021). This could not be confirmed assessing players’ performance with the BALPAI. In contrast, the descriptive statistics of the present study suggest that non-selected PF/C outperformed selected players in passing (see Table 2). In this context, it should be noted that Power Forwards are the positional group that has shown the fastest growth in versatility in the last decade (Rangel et al., 2019). Accordingly, this suggested contradictory performance-related differences may be due to the grouping of the two playing positions (i.e., Power Forward and Center) within this study.

Additionally, compared to SG/SF and PF/C, stronger effects in the expected direction were demonstrated within the group of PG (0.46 ≤ Φ ≤ 0.60, see Table 2). Therefore, these results indicate that selected PG can be identified more clearly than players in other positions based on the performance data assessed with the BALPAI.

#### Position-Dependent Differences

With respect to position-dependent differences, it was hypothesized that PG would be more involved in total ball-bound actions, passing, and dribbling than both SG/SF and PF/C (H2a). Further, it was expected that PG would perform better in passing and dribbling actions than players in the other positional groups (H2b). Moreover, it was assumed that SG/SF demonstrate higher involvement and performance in shooting actions compared to both PG and PF/C (H2c). The results indicate diagnostic validity regarding *offensive game involvement* as PG were more involved in passing, dribbling, and total ball-bound actions than SG/SF and PF/C (H2a). These findings are in line with former research of position-dependent differences in activity demands demonstrating that Guards are more involved in movements with the ball, especially in passing and dribbling (Abdelkrim et al., 2007; Scanlan et al., 2011, 2012; Delextrat et al., 2015; Ferioli et al., 2020a). Further, the results match with those of Ortega et al. (2006), who found that PG made more passes compared to other playing positions in Spanish youth basketball. However, SG/SF were surprisingly not showing higher offensive game involvement with respect to shooting (H2c). It has been reported by research that SG and SF attempt more shots from 3-point range while PF and C take more shots from 2-point range (Sampaio et al., 2006; García-Rubio et al., 2019; Escudero-Tena et al., 2021; Kokanauskas et al., 2021). As BALPAI does not differentiate between shooting ranges, this study thus might not have been able to distinguish between SG/SF and other playing positions with respect to their involvement in shooting actions.

Advantages in *performance* for PG in passing and dribbling compared to the other positional groups were not detected in this study (H2b). Also, SG/SF did not outperform PG or PF/C as far as shooting is concerned (H2c). With respect to passing, this is confirmed by Hatem et al. (2020) who also did not find advantages for Guards with respect to passing. However, previous research analyzing position-dependent differences in game-related statistics have reported advantages for point guards in assists (Sampaio et al., 2006; García-Rubio et al., 2019; Escudero-Tena et al., 2021; Kokanauskas et al., 2021). In contrast to the results of the present study, Hatem et al. (2020) were able to demonstrate a higher proportion of appropriate dribbling actions for Guards. Surprisingly, they also detected a better performance in shooting for Centers. This can be explained by Centers taking a high number of shots close to the basket which are usually executed with high efficiency (e.g., dunks, Kokanauskas et al., 2021). As the observational instrument utilized in that study (i.e., IAD-BB; Folle et al., 2014) does not account for shooting ranges either, centers advanced in this category.

The limited sensitivity to differentiate performance in the present study may be explained by the criteria that has been developed for students or youth basketball players on the entry level (Ibanez et al., 2019). Moreover, the instrument evaluates all players according to the same criteria, regardless of their playing position. However, players have different responsibilities in their respective playing position which requires a more differentiated analysis (Trninić and Dizdar, 2000; Trninić et al., 2000). Therefore, also players aside from the PG who are less skilled with respect to certain ball-bound actions (e.g., passing or dribbling) were able to score high. Further, differences between players in the same playing position should be considered. Although they have to fulfill the same tasks in certain areas, they may have different strengths. In the process of building a team (e.g., youth national team), coaches consider that players complement each other in terms of the various tasks on the basketball court (Pérez-Toledano et al., 2019). As the selection tournament under investigation represented the first stage of selection on national level in Germany, different types of players may have been selected for the same playing positions for further talent development purposes. For example, besides very strong PG “on the ball” (e.g., strong passers and dribblers), also players who rather have outstanding defensive qualities may have been selected. However, as only ball-bound actions in offense were evaluated in the present study, this diversity could not be displayed and players’ performance was not discriminated as expected. In addition, the focus in younger age groups is more on general and less on position-specific skill development (DiFiori et al., 2018; Arede et al., 2019a; Koopmann et al., 2020). Youth basketball players start to specialize in one position at the age of 16 years (Dezman et al., 2001). Assuming that the respective coaches of the investigated players implemented these guidelines and emphasized general skills development throughout their promotion, the players did not have a fixed playing position yet when the study was conducted. Rather, the players may have been used in different playing positions during the selection tournament. This may have affected the differentiation between the playing positions in this study.

The additional comparisons, not utilized for diagnostic validation, revealed that PG outperformed PF/C overall and especially regarding decision making with medium effect sizes (all *p* < 0.05, 0.48 ≤ Φ ≤ 0.49; see Table 3). Performance-related differences may have been identified in these variables because only ball-bound actions were evaluated in this study and PG have more “on-ball tasks” (e.g., passing the ball) than players on the other positions. These findings correspond to the differences in the requirement profiles that are more pronounced between PG and PF/C than among PG and SG/SF (Trninić and Dizdar, 2000; Trninić et al., 2000). This is also indicated by the results of the position-dependent analyses (H2a) demonstrating that the differences between PG and PF/C were more pronounced as reflected in the stronger effect sizes found for the involvement in passing, dribbling, and total ball-bound actions (PG vs. PF/C: 0.53 ≤ Φ ≤ 0.69; PG vs. SG/SF: 0.34 ≤ Φ ≤ 0.55). Further, this is also evident when comparing these playing positions with respect to game-related statistics (Escudero-Tena et al., 2021; Kokanauskas et al., 2021) and physical and physiological demands (Stojanović et al., 2018).

### Limitations and Implications for Optimization

Based on the evaluation of the BALPAI, several limitations need to be addressed in order to derive implications for the optimization of the observational instrument.

*First*, within the present study, only ball-bound actions in offense were considered. Thus, players with more tasks in defense or “off the ball” in offense were possibly disadvantaged by being evaluated according to factors that are not the primary determinants of performance in their respective playing positions. Therefore, the results indicate that only focusing on certain items of the BALPAI in offense when analyzing competitive youth basketball players in different playing positions is not recommended. Rather, the criteria should be weighted with respect to the position-specific requirements in both offense and defense as proposed by Trninić and Dizdar (2000). A system of weighted criteria per position adapted to the BALPAI can contribute to a higher diagnostic validity of the BALPAI when applied in a competitive setting.

*Second*, all players were rated according to the same criteria, which were not adjusted to the performance level or playing position. This may have led to the fact that performance-related differences could barely be detected. Considering sharper and position-specific criteria in future studies could improve the sensitivity of the instrument. For example, a player receives the highest possible rating (i.e., three points) for decision making in a passing action when delivering the ball to a teammate without high defensive pressure and when not having the opportunity to shoot or advance to the basket (see Annex 1; Ibanez et al., 2019). However, when evaluating elite youth basketball players in this context, the criterion should also address whether the pass was the best option if more teammates were available to receive this pass. An adjustment of the rating in this case (i.e., three points if it was the best option, otherwise only two points if the other criteria were met) could contribute to a clearer discrimination between different performance levels (e.g., between selected and non-selected players).

*Third*, as players of different teams were analyzed and compared within this study, the data regarding their game involvement were normalized according to the total number of actions of their respective teams. However, players receive different playing times within their teams, which is determined by the coaching staff based on their performance. The data of this study were not normalized for individual playing time as a selection tournament was analyzed. Here, the main focus was not on winning, but on the presentation of all players, so that equal playing times were assumed. However, the normalization for playing time should be considered when applying the instrument to other competitive settings in future studies (e.g., Ferioli et al., 2020b).

*Fourth*, the impact of intra-individual factors such as the biological maturity status of the players were not considered within the evaluation of players’ performance in this study. However, it has been shown in youth basketball that players’ performance and selection procedures are affected by maturation processes (e.g., Arede et al., 2019b, 2021). In the present study, these processes may have influenced players’ performance, the selection procedures, as well as the ratings performed with the BALPAI, all of which should be addressed in future studies. Furthermore, players’ performance is dynamically influenced by the other players on the court (Rico-González et al., 2020). Therefore, future studies should account for the influence of, for example, teammates (e.g., Piette et al., 2011) or defenders (e.g., shooting, Gorman and Maloney, 2016; dribbling and passing, Vencúrik et al., 2021). Besides, other contextual factors such as the remaining game time or the current score when a play action takes place should be considered. These variables potentially cause increased pressure on players and may affect their performance (Christmann et al., 2018).

## Conclusion

In conclusion, the results of this evaluation confirm the inter-rater reliability while establishing diagnostic validity only for specific variables. Thus, the findings indicate that the instrument, in its current form, is not yet applicable to competitive youth basketball players. This highlights the importance of optimizing the BALPAI for reliable and valid performance assessments of competitive youth basketball players. Future studies should investigate the application of stricter and position-specific criteria to utilize the instrument for talent identification and development purposes.

## Data Availability Statement

The raw data supporting the conclusions of this article will be made available by the authors, without undue reservation.

## Ethics Statement

The studies involving human participants were reviewed and approved by Ethics Committee of the Faculty of Economics and Social Sciences at the University of Tübingen. Written informed consent for participation was not provided by the participants’ legal guardians/next of kin because all data processed in this study were publicly available. Further, the implementation of the study was approved by the German Basketball Federation (Deutscher Basketball Bund, DBB).

## Author Contributions

DR, MS, DL, and OH: conceptualization. DR and MS: data curation, investigation, and validation. DR: formal analysis, resources, and writing—original draft. DR, DL, and OH: methodology and project administration. DL and OH: supervision. DR and DL: visualization. DR, MS, DL, SI, and OH: writing—review and editing. All authors contributed to the article and approved the submitted version.

## Funding

This research received no external funding. We acknowledge support by Open Access Publishing Fund of University of Tübingen and the Aid for Research Groups (GR21149) from the Regional Government of Extremadura (Department of Economy, Science and Digital Agenda), with a contribution from the European Union from the European Funds for Regional Development. The funding supported the payment of the publication fee.

## Conflict of Interest

The authors declare that the research was conducted in the absence of any commercial or financial relationships that could be construed as a potential conflict of interest.

## Publisher’s Note

All claims expressed in this article are solely those of the authors and do not necessarily represent those of their affiliated organizations, or those of the publisher, the editors and the reviewers. Any product that may be evaluated in this article, or claim that may be made by its manufacturer, is not guaranteed or endorsed by the publisher.

## References

[ref1] Abdelkrim N. B. El Fazaa S. El Ati J. (2007). Time–motion analysis and physiological data of elite under-19-year-old basketball players during competition. Br. J. Sports Med. 41, 69–75. doi: 10.1136/bjsm.2006.032318, PMID: 17138630 PMC2658931

[ref2] Ackerman P. L. (2014). Nonsense, common sense, and science of expert performance: talent and individual differences. Intelligence 45, 6–17. doi: 10.1016/j.intell.2013.04.009

[ref3] Arede J. Esteves P. Ferreira A. P. Sampaio J. Leite N. (2019a). Jump higher, run faster: effects of diversified sport participation on talent identification and selection in youth basketball. J. Sports Sci. 37, 2220–2227. doi: 10.1080/02640414.2019.1626114, PMID: 31164046

[ref4] Arede J. Fernandes J. Moran J. Norris J. Leite N. (2021). Maturity timing and performance in a youth national basketball team: do early-maturing players dominate? Int. J. Sports Sci. Coach. 16, 722–730. doi: 10.1177/1747954120980712

[ref5] Arede J. Ferreira A. P. Gonzalo-Skok O. Leite N. (2019b). Maturational development as a key aspect in physiological performance and national-team selection in elite male basketball players. Int. J. Sports Physiol. Perform. 14, 902–910. doi: 10.1123/ijspp.2018-0681, PMID: 30569768

[ref6] Arias J. L. Castejón F. J. (2012). Review of the instruments most frequently employed to assess tactics in physical education and youth sports. J. Teach. Phys. Educ. 31, 381–391. doi: 10.1123/jtpe.31.4.381

[ref7] Baghurst T. Lackman J. Drewson S. Spittler P. Turcott R. Smith M.. (2021). A hot mess: basketball coaches’ perceptions of ability versus actual performances of their athletes. AUC Kinanthropol. 57, 11–25. doi: 10.14712/23366052.2021.3

[ref8] Barquero-Ruiz C. Arias-Estero J. L. Kirk D. (2020). Assessment for tactical learning in games: a systematic review. Eur. Phys. Educ. Rev. 26, 827–847. doi: 10.1177/1356336x19889649

[ref9] Berri D. J. Brook S. L. Fenn A. J. (2011). From college to the pros: predicting the NBA amateur player draft. J. Prod. Anal. 35, 25–35. doi: 10.1007/s11123-010-0187-x

[ref10] Bianchi F. Facchinetti T. Zuccolotto P. (2017). Role revolution: towards a new meaning of positions in basketball. Elect. J. Appl. Stat. Anal. 10, 712–734. doi: 10.1285/i20705948v10n3p712

[ref11] Buekers M. Borry P. Rowe P. (2015). Talent in sports. Some reflections about the search for future champions. Mov. Sport Sc. 88, 3–12. doi: 10.1051/sm/2014002

[ref12] Butterworth A. O’Donoghue P. Cropley B. (2013). Performance profiling in sports coaching: a review. Int. J. Perform. Anal. Sport 13, 572–593. doi: 10.1080/24748668.2013.11868672

[ref13] Byrt T. Bishop J. Carlin J. B. (1993). Bias, prevalence and kappa. J. Clin. Epidemiol. 46, 423–429. doi: 10.1016/0895-4356(93)90018-V 8501467

[ref14] Chen W. Hendricks K. Zhu W. (2013). Development and validation of the basketball offensive game performance instrument. J. Teach. Phys. Educ. 32, 100–109. doi: 10.1123/jtpe.32.1.100

[ref15] Choi D.-H. Kim S.-M. Lee J.-W. Suh S.-H. So W.-Y. (2015). Winning factors: how players’ positional offensive and defensive skills affect probability of victory in the Korea basketball league. Int. J. Sports Sci. Coach. 10, 453–459. doi: 10.1260/1747-9541.10.2-3.453

[ref16] Christmann J. Akamphuber M. Müllenbach A. L. Güllich A. (2018). Crunch time in the NBA – the effectiveness of different play types in the endgame of close matches in professional basketball. Int. J. Sports Sci. Coach. 13, 1090–1099. doi: 10.1177/1747954118772485

[ref17] Clemente F. M. Martins F. M. L. Kalamaras D. Mendes R. S. (2015). Network analysis in basketball: inspecting the prominent players using centrality metrics. J. Physic. Educ. Sport 15, 212–217. doi: 10.7752/jpes.2015.02033

[ref18] Cohen J. (1968). Weighted kappa: nominal scale agreement with provision for scaled disagreement or partial credit. Psychol. Bull. 70, 213–220. doi: 10.1037/h0026256 19673146

[ref19] Cohen J. (1992). A power primer. Psychol. Bull. 112, 155–159. doi: 10.1037//0033-2909.112.1.155, PMID: 19565683

[ref20] Cormery B. Marcil M. Bouvard M. (2008). Rule change incidence on physiological characteristics of elite basketball players: a 10-year-period investigation. Br. J. Sports Med. 42, 25–30. doi: 10.1136/bjsm.2006.033316, PMID: 17526624

[ref21] Csataljay G. O’Donoghue P. Hughes M. Dancs H. (2009). Performance indicators that distinguish winning and losing teams in basketball. Int. J. Perform. Anal. Sport 9, 60–66. doi: 10.1080/24748668.2009.11868464

[ref22] Cui Y. Liu F. Bao D. Liu H. Zhang S. Gómez M.-Á. (2019). Key anthropometric and physical determinants for different playing positions during National Basketball Association Draft Combine Test. Front. Psychol. 10:2359. doi: 10.3389/fpsyg.2019.02359, PMID: 31708831 PMC6820507

[ref23] de la Rubia Riaza A. Lorenzo Calvo J. Mon-López D. Lorenzo A. (2020). Impact of the relative age effect on competition performance in basketball: a qualitative systematic review. Int. J. Environ. Res. Public Health 17:8596. doi: 10.3390/ijerph17228596, PMID: 33228103 PMC7699389

[ref24] Delextrat A. Badiella A. Saavedra V. Matthew D. Schelling X. Torres-Ronda L. (2015). Match activity demands of elite Spanish female basketball players by playing position. Int. J. Perform. Anal. Sport 15, 687–703. doi: 10.1080/24748668.2015.11868824

[ref25] Deutscher Basketball Bund (2020). Bundesjugendlager 2020: Die Nominierten [National Youth Camp 2020: The Nominees] [Online]. Available at: https://www.basketball-bund.de/news/bundesjugendlager-2020-die-nominierten-1111544 (Accessed October 22, 2020).

[ref26] Dezman B. Trninic S. Dizdar D. (2001). Expert model of decision-making system for efficient orientation of basketball players to positions and roles in the game – empirical verification. Coll. Antropol. 25, 141–152. 11787538

[ref27] DiFiori J. P. Güllich A. Brenner J. S. Côté J. Hainline B. Ryan E.. (2018). The NBA and youth basketball: recommendations for promoting a healthy and positive experience. Sports Med. 48, 2053–2065. doi: 10.1007/s40279-018-0950-0, PMID: 29961207 PMC6096539

[ref28] Escudero-Tena A. Rodríguez-Galán V. García-Rubio J. Ibáñez S. J. (2021). Influence of the specific position on the final result of the match in professional basketball. Rev. Psicol. Deport. 30, 19–24.

[ref29] Ferioli D. Rampinini E. Martin M. Rucco D. Torre A. L. Petway A.. (2020a). Influence of ball possession and playing position on the physical demands encountered during professional basketball games. Biol. Sport 37, 269–276. doi: 10.5114/biolsport.2020.95638, PMID: 32879549 PMC7433335

[ref30] Ferioli D. Schelling X. Bosio A. La Torre A. Rucco D. Rampinini E. (2020b). Match activities in basketball games: comparison between different competitive levels. J. Strength Cond. Res. 34, 172–182. doi: 10.1519/jsc.0000000000003039, PMID: 30741861

[ref31] Folle A. Quinaud R. T. B. Barroso M. L. C. Rocha J. C. S. Ramos V. do Nascimento J. V. (2014). Construção e validação preliminar de instrumento de avaliação do desempenho técnico-tático individual no basquetebol. J. Phys. Educ. 25:405. doi: 10.4025/reveducfis.v25i3.23085

[ref32] Gál-Pottyondy A. Petró B. Czétényi A. Négyesi J. Nagatomi R. Kiss R. M. (2021). Collection and advice on basketball field tests—a literature review. Appl. Sci. 11:8855. doi: 10.3390/app11198855

[ref33] García J. Ibáñez S. J. De Santos R. M. Leite N. Sampaio J. (2013). Identifying basketball performance indicators in regular season and playoff games. J. Hum. Kinet. 36, 161–168. doi: 10.2478/hukin-2013-0016, PMID: 23717365 PMC3661887

[ref34] García J. Ibáñez J. S. Gómez A. M. Sampaio J. (2014). Basketball game-related statistics discriminating ACB league teams according to game location, game outcome and final score differences. Int. J. Perform. Anal. Sport 14, 443–452. doi: 10.1080/24748668.2014.11868733

[ref35] García-Ceberino J. M. Gamero M. G. Feu S. Ibáñez S. J. (2020). Differences in technical and tactical learning of football according to the teaching methodology: a study in an educational context. Sustainability 12:6554. doi: 10.3390/su12166554

[ref36] García-Rubio J. Carreras D. Feu S. Antunez A. Ibáñez S. J. (2020). Citius, Altius, Fortius; is it enough to achieve success in basketball? Int. J. Environ. Res. Public Health 17:7355. doi: 10.3390/ijerph17207355, PMID: 33050131 PMC7601684

[ref37] García-Rubio J. Courel-Ibáñez J. Gonzalez-Espinosa S. Ibáñez Godoy S. J. (2019). La especialización en baloncesto: análisis de perfiles de rendimiento en función del puesto específico en etapas de formación [Specialization in basketball: performance profiling analysis according to players’ specific position in formative stages]. Rev. Psicol. Deport. 28, 0132–0139.

[ref38] González-Espinosa S. Mancha-Triguero D. García Santos D. Feu Molina S. Ibáñez Godoy S. J. (2019). Diferencia en el aprendizaje del baloncesto según el género y metodología de enseñanza. Rev. Psicol. Deport. 28, 0086–0092.

[ref39] González-Espinosa S. Molina S. F. García-Rubio J. Medina A. A. García-Santos D. (2017). Diferencias en el aprendizaje según el método de enseñanza-aprendizaje en el baloncesto. Rev. Psicol. Deport. 26, 65–70.

[ref40] Gorman A. D. Maloney M. A. (2016). Representative design: does the addition of a defender change the execution of a basketball shot? Psychol. Sport Exerc. 27, 112–119. doi: 10.1016/j.psychsport.2016.08.003

[ref41] Gréhaigne J.-F. Godbout P. Bouthier D. (1997). Performance assessment in team sports. J. Teach. Phys. Educ. 16, 500–516. doi: 10.1123/jtpe.16.4.500

[ref42] Guimarães E. Baxter-Jones A. Maia J. Fonseca P. Santos A. Santos E.. (2019). The roles of growth, maturation, physical fitness, and technical skills on selection for a Portuguese under-14 years basketball team. Sports 7:61. doi: 10.3390/sports7030061, PMID: 30857137 PMC6473367

[ref43] Hallgren K. A. (2012). Computing inter-rater reliability for observational data: an overview and tutorial. Tutor. Quant. Methods Psychol. 8, 23–34. doi: 10.20982/tqmp.08.1.p023, PMID: 22833776 PMC3402032

[ref44] Hatem A. Folle A. Maciel L. Krapp do Nascimento R. Salles W. Vieira J. (2020). Technical-tactical performance in basketball: evaluation of gaming actions according to specific positions. Motriz. 26, 1–9. doi: 10.1590/s1980-65742020000110200174

[ref45] Höner O. Murr D. Larkin P. Schreiner R. Leyhr D. (2021). Nationwide subjective and objective assessments of potential talent predictors in elite youth soccer: an investigation of prognostic validity in a prospective study. Front. Sports Act. Living 3:638227. doi: 10.3389/fspor.2021.638227, PMID: 34124654 PMC8193982

[ref46] Ibáñez S. J. Feu S. (2021). “Evolución de la investigación académica sobre baloncesto [Evolution of academic research on basketball],” in Una mirada hacia la investigación e innovación sobre baloncesto [A look at basketball research and innovation]. eds. Ibáñez S. J. Feu S. (Cáceres: Universidad de Extremadura, Servicio de Publicaciones), 13–32.

[ref47] Ibanez S. J. Martinez-Fernandez S. Gonzalez-Espinosa S. Garcia-Rubio J. Feu S. (2019). Designing and validating a Basketball Learning and Performance Assessment Instrument (BALPAI). Front. Psychol. 10:1595. doi: 10.3389/fpsyg.2019.01595, PMID: 31354590 PMC6635796

[ref48] Johnston K. Baker J. (2020). Waste reduction strategies: factors affecting talent wastage and the efficacy of talent selection in sport. Front. Psychol. 10:2925. doi: 10.3389/fpsyg.2019.02925, PMID: 31998188 PMC6967295

[ref49] Johnston K. Farah L. Baker J. (2021). Storm clouds on the horizon: on the emerging need to tighten selection policies. Front. Sports Act. Living 3:772181. doi: 10.3389/fspor.2021.772181, PMID: 34805981 PMC8600356

[ref50] Johnston K. Wattie N. Schorer J. Baker J. (2018). Talent identification in sport: a systematic review. Sports Med. 48, 97–109. doi: 10.1007/s40279-017-0803-2, PMID: 29082463

[ref51] Kalén A. Lundkvist E. Ivarsson A. Rey E. Pérez-Ferreirós A. (2021). The influence of initial selection age, relative age effect and country long-term performance on the re-selection process in European basketball youth national teams. J. Sports Sci. 39, 388–394. doi: 10.1080/02640414.2020.1823109, PMID: 32996408

[ref52] Kalén A. Pérez-Ferreirós A. Rey E. Padrón-Cabo A. (2017). Senior and youth national team competitive experience: influence on player and team performance in European basketball championships. Int. J. Perform. Anal. Sport 17, 832–847. doi: 10.1080/24748668.2017.1405610

[ref53] Kokanauskas O. Bietkis T. Arede J. Leite N. (2021). Modelling youth basketball performance profile in European championships. Rev. Psicol. Deport. 30, 258–262.

[ref54] König S. Heckel J. (2021). “Match analysis in basketball,” in Match Analysis: How to Use Data in Professional Sport. ed. Memmert D. (Abingdon: Routledge), 51–58.

[ref55] Koopmann T. Faber I. Baker J. Schorer J. (2020). Assessing technical skills in talented youth athletes: a systematic review. Sports Med. 50, 1593–1611. doi: 10.1007/s40279-020-01299-4, PMID: 32495253 PMC7441090

[ref56] Korte F. Lames M. (2018). Characterizing different team sports using network analysis. Curr. Issues Sport Sci. 3:005. doi: 10.15203/CISS_2018.005

[ref57] Landis J. R. Koch G. G. (1977). The measurement of observer agreement for categorical data. Biometrics 33, 159–174. doi: 10.2307/2529310 843571

[ref58] Lath F. Koopmann T. Faber I. Baker J. Schorer J. (2021). Focusing on the coach’s eye; towards a working model of coach decision-making in talent selection. Psychol. Sport Exerc. 56:102011. doi: 10.1016/j.psychsport.2021.102011

[ref59] Leicht A. S. Gómez M. A. Woods C. T. (2017). Explaining match outcome during the men’s basketball tournament at the Olympic games. J. Sports Sci. Med. 16, 468–473. 29238245 PMC5721175

[ref60] Lorenzo A. Gómez M. Á. Ortega E. Ibáñez S. J. Sampaio J. (2010). Game related statistics which discriminate between winning and losing under-16 male basketball games. J. Sports Sci. Med. 9, 664–668. 24149794 PMC3761811

[ref61] Milanovic D. Stefan L. Sporis G. Vuleta D. Selmanovic A. (2016). Effects of situational efficiency indicators on final outcome among male basketball teams on the Olympic games in London 2012. Acta Kinesiolog. 10, 78–84.

[ref62] Muñoz J. Gamonales J. M. León K. Ibáñez S. J. (2018). Formación de codificadores y fiabilidad de los registros. Una aplicación al goalball [Training of Coders and Reliability. An Application to the Goalball]. Rev. Int. Med. Cienc. Act. Física Deport. 18, 669–691. doi: 10.15366/rimcafd2018.72.005

[ref63] NBBL gGmbH (2020). JBBL Teams [Online]. Available at: https://www.nbbl-basketball.de/teams/jbbl-teams/ (Accessed October 22, 2020).

[ref64] Okazaki V. H. A. Rodacki A. L. F. Satern M. N. (2015). A review on the basketball jump shot. Sports Biomech. 14, 190–205. doi: 10.1080/14763141.2015.1052541, PMID: 26102462

[ref65] Ortega E. Cárdenas D. Sainz de Baranda P. Palao J. (2006). Analysis of the final actions used in basketball during formative years according to player’s position. J. Hum. Mov. Stud. 50, 421–437.

[ref66] Oslin J. L. Mitchell S. A. Griffin L. L. (1998). The game performance assessment instrument (GPAI): development and preliminary validation. J. Teach. Phys. Educ. 17, 231–243. doi: 10.1123/jtpe.17.2.231

[ref67] Pérez-Toledano M. Á. Rodriguez F. J. García-Rubio J. Ibañez S. J. (2019). Players’ selection for basketball teams, through performance index rating, using multiobjective evolutionary algorithms. PLoS One 14:e0221258. doi: 10.1371/journal.pone.0221258, PMID: 31483835 PMC6726145

[ref68] Piette J. Pham L. Anand S. (2011). “Evaluating basketball player performance via statistical network modeling,” in *The 5th MIT Sloan Sports Analytics Conference*. March 4-5, 2011; Boston, MA: USA.

[ref69] Rangel W. Ugrinowitsch C. Lamas L. (2019). Basketball players’ versatility: assessing the diversity of tactical roles. Int. J. Sports Sci. Coach. 14, 552–561. doi: 10.1177/1747954119859683

[ref70] Rico-González M. Pino-Ortega J. Nakamura F. Y. Moura F. A. Los Arcos A. (2020). Identification, computational examination, critical assessment and future considerations of distance variables to assess collective tactical behaviour in team invasion sports by positional data: a systematic review. Int. J. Environ. Res. Public Health 17:1952. doi: 10.3390/ijerph17061952, PMID: 32192000 PMC7143020

[ref71] Roberts A. H. Greenwood D. A. Stanley M. Humberstone C. Iredale F. Raynor A. (2019). Coach knowledge in talent identification: a systematic review and meta-synthesis. J. Sci. Med. Sport 22, 1163–1172. doi: 10.1016/j.jsams.2019.05.008, PMID: 31133481

[ref72] Rogers M. Crozier A. J. Schranz N. K. Eston R. G. Tomkinson G. R. (2021). Player profiling and monitoring in basketball: a Delphi study of the most important non-game performance indicators from the perspective of elite athlete coaches. Sports Med. 1–13. doi: 10.1007/s40279-021-01584-w, PMID: 34739718

[ref73] Rösch D. Deutsch Q. Höner O. (2021). Scoutingdaten im Nachwuchs-basketball. Zusammenhang mit dem Erfolg im Erwachsenenalter [Scouting data in junior basketball and its connection to success in adulthood]. Leistungssport 51, 45–49.

[ref74] Russell J. L. McLean B. D. Impellizzeri F. M. Strack D. S. Coutts A. J. (2021). Measuring physical demands in basketball: an explorative systematic review of practices. Sports Med. 51, 81–112. doi: 10.1007/s40279-020-01375-9, PMID: 33151481

[ref75] Sampaio J. Ibáñez S. J. Lorenzo A. (2013). “Basketball,” in Routledge Handbook of Sports Performance Analysis. eds. McGarry T. O’Donoghue P. Sampaio J. (London, UK: Routledge) 357–366.

[ref76] Sampaio J. Janeira M. Ibáñez S. J. Lorenzo A. (2006). Discriminant analysis of game-related statistics between basketball guards, forwards and centres in three professional leagues. Eur. J. Sport Sci. 6, 173–178. doi: 10.1080/17461390600676200

[ref77] Saward C. Morris J. G. Nevill M. E. Sunderland C. (2019). The effect of playing status, maturity status, and playing position on the development of match skills in elite youth football players aged 11–18 years: a mixed-longitudinal study. Eur. J. Sport Sci. 19, 315–326. doi: 10.1080/17461391.2018.1508502, PMID: 30115002

[ref78] Scanlan A. Dascombe B. Reaburn P. (2011). A comparison of the activity demands of elite and sub-elite Australian men’s basketball competition. J. Sports Sci. 29, 1153–1160. doi: 10.1080/02640414.2011.582509, PMID: 21777151

[ref79] Scanlan A. T. Dascombe B. J. Reaburn P. Dalbo V. J. (2012). The physiological and activity demands experienced by Australian female basketball players during competition. J. Sci. Med. Sport 15, 341–347. doi: 10.1016/j.jsams.2011.12.008, PMID: 22244965

[ref80] Schorer J. Faber I. Koopmann T. Büsch D. Baker J. (2020). Predictive value of coaches’ early technical and tactical notational analyses on long-term success of female handball players. J. Sports Sci. 38, 2208–2214. doi: 10.1080/02640414.2020.1776923, PMID: 32516095

[ref81] Schweizer G. Furley P. Rost N. Barth K. (2020). Reliable measurement in sport psychology: the case of performance outcome measures. Psychol. Sport Exerc. 48:101663. doi: 10.1016/j.psychsport.2020.101663

[ref82] Stojanović E. Stojiljković N. Scanlan A. T. Dalbo V. J. Berkelmans D. M. Milanović Z. (2018). The activity demands and physiological responses encountered during basketball match-play: a systematic review. Sports Med. 48, 111–135. doi: 10.1007/s40279-017-0794-z, PMID: 29039018

[ref83] Teramoto M. Cross C. L. Rieger R. H. Maak T. G. Willick S. E. (2018). Predictive validity of National Basketball Association Draft Combine on future performance. J. Strength Cond. Res. 32, 396–408. doi: 10.1519/jsc.0000000000001798, PMID: 28135222

[ref84] Till K. Baker J. (2020). Challenges and [possible] solutions to optimizing talent identification and development in sport. Front. Psychol. 11:664. doi: 10.3389/fpsyg.2020.00664, PMID: 32351427 PMC7174680

[ref85] Torres-Unda J. Zarrazquin I. Gil J. Ruiz F. Irazusta A. Kortajarena M.. (2013). Anthropometric, physiological and maturational characteristics in selected elite and non-elite male adolescent basketball players. J. Sports Sci. 31, 196–203. doi: 10.1080/02640414.2012.725133, PMID: 23046359

[ref86] Trninić S. Dizdar D. (2000). System of the performance evaluation criteria weighted per positions in the basketball game. Coll. Antropol. 24, 217–234. 10895549

[ref87] Trninić S. Dizdar D. Dežman B. (2000). Empirical verification of the weighted system of criteria for the elite basketball players quality evaluation. Coll. Antropol. 24, 443–465. 11216413

[ref88] Trunić N. Mladenović M. (2014). The importance of selection in basketball. Sport Sci. Pract. 4, 65–81.

[ref89] Vannest K. J. Parker R. I. Gonen O. Adiguzel T. (2016). Single Case Research: web based calculators for scr analysis. (Version 2.0) [Web-based application]. [Online]. College Station, TX: Texas A&M University. Available at: http://singlecaseresearch.org/calculators/pabak-os (Accessed December 5, 2021).

[ref90] Vencúrik T. Nykodým J. Bokůvka D. Rupčić T. Knjaz D. Dukarić V.. (2021). Determinants of dribbling and passing skills in competitive games of women’s basketball. Int. J. Environ. Res. Public Health 18:1165. doi: 10.3390/ijerph18031165, PMID: 33525670 PMC7908611

