# Supplementary material for: Performance Differences in Male Youth Basketball Players According to Selection Status and Playing Position: An Evaluation of the Basketball Learning and Performance Assessment Instrument
Source: Front Psychol. 2022 May 6;13:859897. doi: 10.3389/fpsyg.2022.859897 (PMC9121897; doi:10.3389/fpsyg.2022.859897)
Supplement: Supplementary file 1 [file Table_1.DOCX]

**Supplementary Materials**

**Table 1.** Descriptive statistics for the total ball-bound actions of the sampled teams.

| **Variables** | ***M* ± *SD*** |
| --- | --- |
| All Actions | 434.00 ± 69.77 |
| Shooting | 64.60 ± 6.07 |
| Passing | 117.40 ± 29.31 |
| Dribbling | 127.20 ± 20.51 |
| Receiving | 124.80 ± 26.49 |

*N* = 5.
