# Supplementary material for: Performance Differences in Male Youth Basketball Players According to Selection Status and Playing Position: An Evaluation of the Basketball Learning and Performance Assessment Instrument
Source: Front Psychol. 2022 May 6;13:859897. doi: 10.3389/fpsyg.2022.859897 (PMC9121897; doi:10.3389/fpsyg.2022.859897)
Supplement: Supplementary file 2 [file Table_2.DOCX]

**Supplementary Materials**

**Table 2.** Videos of the U15 national selection tournament.

| **No.** | **Date** | **Data availability** |
| --- | --- | --- |
| 1 | October 2, 2020 | <https://www.youtube.com/watch?v=6t-eCsiZc5Q> |
| 2 | October 3, 2020 | <https://www.youtube.com/watch?v=lcceeKRSnG8> |
| 3 | October 4, 2020 | <https://www.youtube.com/watch?v=JS2K9AVEWiU> |

**Note.** Access to each of the listed hyperlinks was verified on March 4, 2022.
